# The Relationship Between Preoperative Neutrophil–Lymphocyte Ratio and Postoperative Length of Stay in Carotid Body Tumor Resection

**DOI:** 10.1155/ijog/5431545

**Published:** 2025-04-23

**Authors:** Biao Wu, Jiang Zhu, Liang Chen, Xiaonan Wang, Hao Zhang, Kunyu Guan, Yu Li

**Affiliations:** ^1^Department of Vascular Surgery, Changhai Hospital, Shanghai, China; ^2^Pediatrics, Changhai Hospital, Shanghai, China

**Keywords:** carotid body tumor, neutrophil-to-lymphocyte ratio, postoperative length of stay

## Abstract

Carotid body tumor (CBT) resection is a complex surgical procedure often resulting in extended postoperative length of stay (PLOS) due to potential nerve injuries, arterial damage, and wound complications. The neutrophil-to-lymphocyte ratio (NLR) is a known marker of systemic inflammation and has been associated with adverse outcomes in various surgical settings. However, the relationship between preoperative NLR and PLOS in CBT patients has not been explored. This study aims to investigate the association between preoperative NLR and PLOS in CBT resections, particularly examining whether elevated NLR correlates with longer hospital stays and potentially hinders recovery. In this retrospective cohort study, we analyzed data from 231 CBT patients who underwent resection at Changhai Hospital, Shanghai, between 2008 and 2020. Patients were grouped based on their PLOS (short, medium, and long stays), and NLR was calculated from peripheral blood samples taken preoperatively. Univariate and multivariate regression models adjusted for sociodemographic and operative factors, including Shamblin classification, were used to examine the relationship between NLR and PLOS. Elevated preoperative NLR has been found to be significantly correlated with prolonged PLOS, with each incremental increase in NLR corresponding to an approximate extension of 0.12 days in PLOS after adjusting for confounding factors. Stratified analysis revealed that this association was most pronounced in patients with Shamblin II tumors, likely due to the moderate tumor size and adhesion in these cases, which necessitates more extensive dissection and increases vulnerability to nerve injury. Elevated preoperative NLR may serve as a predictor of prolonged recovery in CBT resections, particularly for Shamblin II cases. This finding highlights the potential utility of NLR in preoperative assessment and patient management to optimize surgical timing and reduce hospital stays. Further research with larger cohorts is needed to confirm the predictive value of NLR and explore its clinical application in surgical planning for CBT patients.

## 1. Introduction

Carotid body tumor (CBT) is a slow-growing, benign, and rare tumor in the neck region, originating from the paraganglion cells of the carotid body [1]. These tumors, while typically benign, can grow to a significant size, posing a risk of compressing surrounding structures such as the neck's nerves and carotid artery, potentially leading to severe complications including stroke [2]. Surgical resection is widely regarded as the most effective treatment for CBTs, with early intervention being strongly recommended by many researchers due to its high success rates, which can range from 50% to 80% [3]. However, due to the tumor's tight adhesion to surrounding structures such as carotid artery and nearby nerves, surgery often results in nerve injuries, arterial damage, and wound complications [4]. Ali et al. reported that arterial injury is frequently accompanied by nerve damage during CBT resections [5].

Inflammation plays a central role in the body's repair mechanisms. While immune cells contribute to neuronal regeneration and functional recovery after injury, excessive inflammation can also cause secondary damage, hindering postinjury nerve repair. The neutrophil-to-lymphocyte ratio (NLR) has been established as a readily available and cost-effective biomarker for systemic inflammation [6, 7]. It has been particularly useful in assessing conditions such as sepsis [8], chronic obstructive pulmonary disease, and abdominal surgeries [9]. An elevated NLR, reflecting neutrophilia and lymphocytopenia in response to systemic inflammation, has been associated with poor outcomes in a range of tumors and cardiovascular diseases [10–13]. This inflammatory response may also contribute to nerve damage or delayed wound healing, potentially extending postoperative length of stay (PLOS).

Considering the extensive healthcare challenges introduced by the COVID-19 pandemic, such as increased hospitalization rates and economic constraints, there has been a growing emphasis on reducing the duration of hospital stays to alleviate the strain on healthcare systems. CBT is frequently associated with chronic hypoxia, and numerous patients arrive from high-altitude regions [14], where healthcare and economic conditions are often less favorable compared to more developed urban areas. Shortening PLOS is a pivotal factor in reducing resource consumption and alleviating financial pressures for patients and healthcare systems.

While NLR is widely recognized as a marker of inflammation, its role in CBT has not yet been explored. This study aims to explore the correlation between NLR and PLOS in patients undergoing CBT resections, with the goal of identifying optimal surgical timing and strategies to minimize hospitalization durations.

## 2. Methods

### 2.1. Study Design and Patients

**Figure 1:**
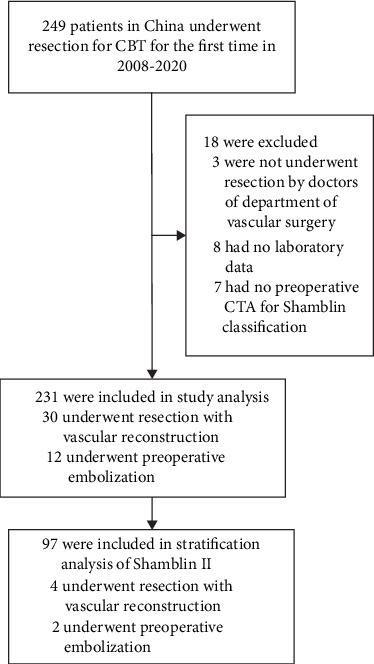
Flow chart of CBT resection included in the study.

This retrospective observational study was conducted in the Department of Vascular Surgery at Changhai Hospital (Shanghai, China). Between November 2008 and December 2020, a total of 231 patients who underwent CBT resection were consecutively included without selection bias. Patients were excluded if they lacked medical records, had no preoperative computed tomography angiography (CTA) confirming Shamblin classification, or did not undergo surgical treatment (see Figure 1). The study was conducted in accordance with the Declaration of Helsinki and approved by the Changhai Hospital Ethics Committee. Informed written consent was obtained from all participants.

### 2.2. Definitions and Data Collection

Patient data were retrospectively retrieved from the hospital's electronic medical record system. PLOS was recorded as a continuous variable but categorized for univariate analysis into three groups: short (1–3 days), medium (4–5 days), and long (≥ 6 days). Peripheral venous blood samples were collected on admission to measure neutrophil and lymphocyte counts, and the NLR was calculated by dividing the neutrophil count by the lymphocyte count.

Surgical details were extracted from anesthesia and operative notes. CBT resections were performed with or without vascular reconstruction, depending on tumor size, stiffness, and adhesion to the carotid artery. Some patients underwent preoperative embolization, as indicated.

### 2.3. Statistical Analysis

Continuous variables were presented as mean ± standard deviation (SD), while categorical variables were expressed as frequencies and percentages. Differences between PLOS groups were tested using the *χ*^2^ test for categorical variables and Student's *t*-tests for continuous variables (both normally and nonnormally distributed). Univariate and multivariate linear regression models were used to assess the relationship between NLR and PLOS.

Three models were constructed: Model I was unadjusted; Model II was adjusted for sociodemographic factors (age, sex, height, weight, body mass index, systolic blood pressure, diastolic blood pressure, pulse pressure, hypertension, diabetes, coronary heart disease, cerebral infarction, alcohol use, and smoking status); and Model III was further adjusted for operative factors, including surgical approach, embolization, and Shamblin classification (Table 1). To account for potential nonlinearity between NLR and PLOS, a smooth curve fitting was applied using a penalized spline method. Subgroup analysis was conducted based on Shamblin classification to evaluate the relationship between NLR and PLOS.

All statistical analyses were performed using R (http://www.R-project.org, The R Foundation) and EmpowerStats (http://www.empowerstats.com, X&Y Solutions, Inc., Boston, Massachusetts). A two-sided *p*-value < 0.05 was considered statistically significant.

## 3. Results

### 3.1. Patient Characteristics

The demographics and clinical characteristics of the 231 patients are summarized in Table 1, stratified by PLOS groups: short (1–3 days), medium (4–5 days), and long (≥ 6 days). The medium PLOS group comprised the largest proportion of patients (31.2%, 44.6%, and 24.2%, respectively). Patients in the short PLOS group had a higher mean age compared to those in the other groups (47.03 ± 12.58 years vs. 41.01 ± 12.82 years vs. 41.84 ± 12.12 years, *p* < 0.01) and a greater pulse pressure (48.01 ± 9.81 mmHg vs. 44.22 ± 8.94 mmHg vs. 42.68 ± 8.70 mmHg, *p* < 0.01). Operational time was significantly longer in the long PLOS group compared to the short and medium groups (130.86 ± 108.03 min vs. 150.58 ± 63.05 min vs. 197.51 ± 80.35 min, *p* < 0.01). Among the patients who underwent resection without vascular reconstruction, there was little a difference in PLOS across the groups (69 vs. 92 vs. 40), but for those who had vascular reconstruction, more patients had long PLOS (3 vs. 11 vs. 16, *p* < 0.01). Shamblin classification was also associated with PLOS; in the short PLOS group, there were equal numbers of Shamblin II and III cases (*n* = 30 each), while Shamblin I cases were fewer (16.67% vs. 41.67% vs. 41.67%). In the medium PLOS group, Shamblin II predominated (14.56% vs. 47.57% vs. 37.86%). For long PLOS, the majority of patients were Shamblin III (*n* = 35, 62.50%).

### 3.2. Laboratory Data

Laboratory data, as shown in Table 2, demonstrated that longer PLOS groups had higher white blood cell (WBC) counts (6.24 ± 1.52 × 10^9^/L vs. 6.82 ± 2.26 × 10^9^/L vs. 7.63 ± 2.84 × 10^9^/L, *p* < 0.01), but lower lymphocyte percentages (30.52 ± 8.66% vs. 30.78 ± 10.66% vs. 26.71 ± 11.47%, *p* = 0.04). Notably, the NLR in the short PLOS group was significantly lower than in the long PLOS group (2.37 ± 2.41 vs. 2.67 ± 2.76 vs. 4.35 ± 5.91, *p* < 0.01). Hemoglobin (Hb) levels also differed significantly between groups (138.03 ± 34.00 g/L vs. 127.67 ± 18.59 g/L vs. 129.55 ± 18.98 g/L, *p* = 0.02).

### 3.3. Univariate Analysis Related to PLOS

Univariate linear regression analysis (Table 3) revealed a positive correlation between NLR and PLOS (*β* = 0.13, 95% CI: 0.07–0.19, *p* < 0.01). Nonoperative approaches compared to traditional surgery (*β* = 1.42, 95% CI: 0.75–2.09, *p* < 0.01) and Shamblin III classification compared to Shamblin I (*β* = 0.98, 95% CI: 0.26–1.71, *p* < 0.01) were also positively associated with longer PLOS.

### 3.4. Multivariate Analysis of NLR and PLOS

Three models were constructed to analyze the independent effect of NLR on PLOS using both univariate and multivariate linear regressions (Table 4). In Model I (unadjusted), NLR was associated with a PLOS increase of 0.13 days for every 1-unit rise in NLR (*β* = 0.13, 95% CI: 0.07–0.19, *p* < 0.01). After adjusting for sociodemographic factors in Model II, the effect size remained significant, with a 0.11-day increase in PLOS per 1-unit NLR increase (*β* = 0.11, 95% CI: 0.05–0.17, *p* < 0.01). In Model III, after further adjusting for operative variables (e.g., surgical method, embolization, and Shamblin classification), the effect size increased slightly, with each 1-unit increase in NLR associated with a 0.12-day longer PLOS (*β* = 0.12, 95% CI: 0.06–0.18, *p* < 0.01).

### 3.5. Curve Fitting of NLR and PLOS

**Figure 2:**
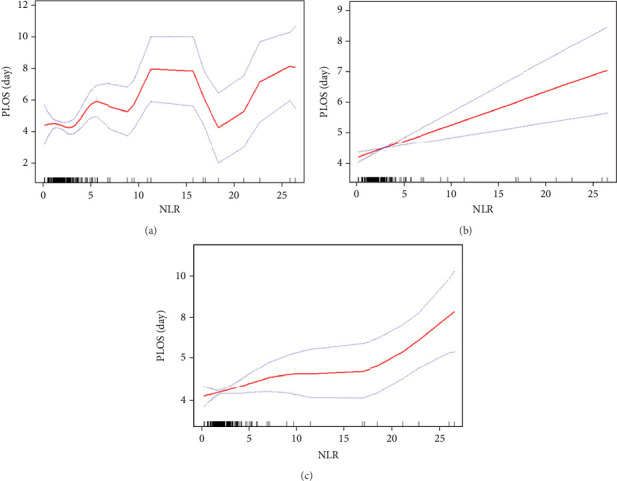
The curve fitting between NLR and PLOS. (a) None was adjusted. (b) Adjusted for gender, age, stature, weight, BMI, systolic pressure, diastolic pressure, pulse pressure, hypertension, diabetes, CHD, cerebrovascular disease, drank, and smoke. (c) Model B additionally adjusted for operation method, embolization, and Shamblin classification.

The relationship between NLR and PLOS was further analyzed using a smooth curve fitting approach (Figure 2). After adjusting for confounders, the relationship between NLR and PLOS was linear, with an effect size of 0.12 days for every 1-unit increase in NLR (*β* = 0.12, 95% CI: 0.06–0.18, *p* < 0.01).

### 3.6. Stratification Analysis by Shamblin Classification

Stratified analysis by Shamblin classification (Table 5) demonstrated that the relationship between NLR and PLOS was statistically significant in Shamblin II patients, with a *β* value of 0.20 (95% CI: 0.09–0.29, *p* < 0.01) after adjusting for all other variables.

## 4. Discussion

Our research revealed a significant correlation between elevated preoperative NLR and prolonged PLOS, particularly among patients with Shamblin II CBTs, aligning with findings from studies on other cancer types. The preoperative NLR, as measured in this study, may indicate an inflammatory response associated with the underlying tumor, rather than postoperative infection, as supported by recent research. This distinction is crucial, as our emphasis was on preoperative inflammatory status, rather than the consequences of postoperative complications such as wound infection or nerve damage, which are unrelated to the preoperative NLR.

Previous studies have linked higher NLR with poor outcomes in various conditions, including cancer and cardiovascular diseases, highlighting its role as a marker of systemic inflammation [15, 16]. In our study, patients with higher preoperative NLR had longer PLOS, supporting the hypothesis that elevated preoperative inflammation may hinder recovery, potentially due to increased vulnerability to nerve injury or delayed healing processes.

We found that the relationship between NLR and PLOS varied across Shamblin classifications. Stratified analysis revealed that NLR had the strongest association with PLOS in Shamblin II patients. This may be explained by the tumor's moderate size and adhesion in Shamblin II cases, which requires more extensive nerve dissection during surgery, increasing the risk of transient nerve injury. Preoperative inflammation, as indicated by elevated NLR, may exacerbate this risk and prolong recovery. In contrast, Shamblin I tumors, being smaller with minimal adhesion, typically involve less surgical complexity and shorter recovery times, making the impact of preoperative inflammation less significant. Shamblin III tumors are larger and more tightly adhered, causing extensive tissue damage during surgery [17]. In these cases, the severity of the procedure may mask the effects of preoperative inflammation, reducing the predictive value of NLR.

In addition to NLR, other factors including pulse pressure, surgical technique, and Shamblin classification were also notably linked to PLOS. Nevertheless, preoperative embolization failed to markedly decrease PLOS, potentially owing to the time consumed by the embolization procedure and the absence of a substantial decline in postoperative complication rates in certain instances [18, 19].

Our findings contribute to the understanding of the relationship between preoperative inflammatory status and recovery in CBT surgeries. While the association between NLR and PLOS is promising, further research is needed to confirm whether preoperative NLR can reliably predict surgical outcomes and guide decision-making, particularly in the context of patient stratification based on tumor characteristics [20].

## 5. Conclusion

The study demonstrated a significant association between preoperative NLR and PLOS, particularly in Shamblin II CBT patients. While NLR may serve as a useful marker for assessing preoperative inflammation, its role in predicting surgical outcomes requires further validation. Future research should concentrate on validating these results in broader populations and investigating the potential application of NLR in optimizing surgical timing and enhancing patient management strategies.

### 5.1. Limitations

Although this study demonstrates a significant relationship between NLR and PLOS, several limitations should be considered: The study specifically targets patients undergoing CBT resection, thereby restricting the broad applicability and generalization of its findings to other patient groups or surgical procedures.

Despite being the largest single-center study to date on this subject, the sample size is still relatively small, which may reduce the statistical power and lead to some results lacking statistical significance. PLOS is influenced by numerous factors beyond inflammation. NLR serves as a significant indicator of preoperative inflammation, yet relying solely on NLR for predicting PLOS has its limitations. Nevertheless, we have endeavors to consider potential confounding factors in our analysis to enhance the reliability of the results.

## Figures and Tables

**Table 1:** Patient characteristic of CBT.

	**PLOS**			**p** **-value**
	**Short PLOS** **(** **n** = 72**)**	**Medium PLOS** **(** **n** = 103**)**	**Long PLOS** **(** **n** = 56**)**	
Patient characteristics				
Gender				0.12
Female	43 (59.72%)	65 (63.11%)	26 (46.43%)	
Male	29 (40.28%)	38 (36.89%)	30 (53.57%)	
Age (year)	47.03 ± 12.58	41.01 ± 12.82	41.84 ± 12.12	**< 0.01**
Stature (cm)	163.24 ± 8.11	164.11 ± 7.02	165.19 ± 7.70	0.40
Weight (kg)	63.24 ± 10.33	61.56 ± 11.65	63.37 ± 11.04	0.50
BMI (kg/m^2^)	23.46 ± 3.42	22.78 ± 3.36	22.76 ± 3.01	0.39
PP (mmHg)	48.01 ± 9.81	44.22 ± 8.94	42.68 ± 8.70	**< 0.01**
Drank				0.27
No	71 (98.61%)	98 (95.15%)	52 (92.86%)	
Yes	1 (1.39%)	5 (4.85%)	4 (7.14%)	
Smoke				0.95
No	66 (91.67%)	93 (90.29%)	51 (91.07%)	
Yes	6 (8.33%)	10 (9.71%)	5 (8.93%)	
Comorbidities				
Hypertension	12	10	5	0.28
Diabetes mellitus	0	6	3	0.12
CHD	0	3	0	0.15
Cerebral infarction	2	1	2	0.51
Tumor characteristics				
Operation side				0.52
Left	38 (52.78%)	59 (57.28%)	35 (62.50%)	
Right	33 (45.83%)	44 (42.72%)	21 (37.50%)	
Both	1 (1.39%)	0 (0.00%)	0 (0.00%)	
Operational time (minute)	130.86 ± 108.03	150.58 ± 63.05	197.51 ± 80.35	**< 0.01**
Operation method				**< 0.01**
Resection with no vascular reconstruction	69 (95.83%)	92 (89.32%)	40 (71.43%)	
Resection with vascular reconstruction	3 (4.17%)	11 (10.68%)	16 (28.58%)	
Embolization				0.37
No	67 (93.06%)	100 (97.09%)	52 (92.86%)	
Yes	5 (6.94%)	3 (2.91%)	4 (7.14%)	
Shamblin classification				**0.03**
I	12 (16.67%)	15 (14.56%)	3 (5.36%)	
II	30 (41.67%)	49 (47.57%)	18 (32.14%)	
III	30 (41.67%)	39 (37.86%)	35 (62.50%)	

*Note:* Data are presented as *n*(%) or mean ± standard deviation (SD) unless stated otherwise.

Abbreviations: BMI = body mass index, CHD = coronary heart disease, PLOS = postoperative length of stay, PP = pulse pressure.

**Table 2:** Laboratory data.

	**Short PLOS** **(** **n** = 72**)**	**Medium PLOS** **(** **n** = 103**)**	**Long PLOS** **(** **n** = 56**)**	**p** ** -value**
WBC (10^9^/L)	6.24 ± 1.52	6.82 ± 2.26	7.63 ± 2.84	**< 0.01**
LYM (%)	30.52 ± 8.66	30.78 ± 10.66	26.71 ± 11.47	**0.04**
NEUT (%)	58.80 ± 11.54	59.77 ± 11.66	63.51 ± 15.82	0.10
RBC (10^12^/L)	4.50 ± 0.64	4.40 ± 0.56	4.43 ± 0.49	0.52
Hb (g/L)	138.03 ± 34.00	127.67 ± 18.59	129.55 ± 18.98	**0.02**
Platelet (10^9^/L)	238.24 ± 59.34	234.36 ± 72.08	240.21 ± 82.54	0.87
DD (mg/L)	0.33 ± 0.17	0.40 ± 0.53	0.33 ± 0.35	0.48
TT (s)	16.22 ± 2.11	16.40 ± 1.35	16.61 ± 1.49	0.44
Fib (g/L)	3.26 ± 0.73	3.67 ± 2.55	3.98 ± 1.48	0.11
FDP (μg/mL)	2.42 ± 1.31	2.21 ± 2.10	2.19 ± 1.99	0.73
TG (mmol/L)	1.60 ± 1.36	1.15 ± 0.68	1.85 ± 3.91	0.18
HDL (mmol/L)	1.23 ± 0.31	2.72 ± 12.71	1.23 ± 0.39	0.53
LDL (mmol/L)	2.59 ± 0.76	2.39 ± 0.80	2.44 ± 0.58	0.33
NLR	2.37 ± 2.41	2.67 ± 2.76	4.35 ± 5.91	**< 0.01**

*Note:* Data are presented as mean ± standard deviation (SD) unless stated otherwise.

Abbreviations: DD = d-dimer, FDP = fibrinogen degradation products, Fib = fibrinogen, Hb = hemoglobin, HDL = high-density lipoprotein, LDL = low-density lipoprotein, LYM = lymphocyte, NEUT = neutrophilic granulocyte, NLR = neutrophil-lymphocyte ratio, RBC = red blood cell, TG = triglyceride, TT = thrombin time, WBC = white blood cell.

**Table 3:** Univariate analysis of CBT.

	**Statistics**	**PLOS**	**p** **-value**
Patient characteristics			
Gender			
Female	134 (58.01%)	0	
Male	97 (41.99%)	0.47 (0.00, 0.94)	0.05
Age (year)	43.09 ± 12.81	−0.02 (−0.03, 0.00)	0.08
BMI (kg/m^2^)	22.99 ± 3.30	−0.05 (−0.12, 0.01)	0.12
PP (mmHg)	45.03 ± 9.36	−0.04 (−0.06, −0.01)	**< 0.01**
Drank			
No	221 (95.67%)	0	
Yes	10 (4.33%)	0.73 (−0.41, 1.87)	0.21
Smoke			
No	210 (90.91%)	0	
Yes	21 (9.09%)	−0.19 (−1.00, 0.62)	0.65
Comorbidities			
Hypertension			
No	204 (88.31%)	0	
Yes	27 (11.69%)	−0.39 (−1.11, 0.34)	0.30
Diabetes mellitus			
No	222 (96.10%)	0	
Yes	9 (3.90%)	0.53 (−0.68, 1.74)	0.39
CHD			
No	228 (98.70%)	0	
Yes	3 (1.30%)	0.40 (−1.66, 2.47)	0.70
Cerebrovascular disease			
No	226 (97.84%)	0	
Yes	5 (2.16%)	−0.21 (−1.81, 1.40)	0.80
Tumor characteristics			
Operation side			
Left	132 (57.14%)	0	
Right	98 (42.42%)	−0.31 (−0.79, 0.16)	0.19
Both	1 (0.43%)	−1.74 (−5.29, 1.81)	0.34
Operation method			
Resection with no vascular reconstruction	201 (87.01%)	0	
Resection with vascular reconstruction	30 (12.99%)	1.42 (0.75, 2.09)	**< 0.01**
Embolization			
No	219 (94.81%)	0	
Yes	12 (5.19%)	−0.02 (−1.07, 1.03)	0.97
Shamblin classification			
I	30 (12.99%)	0	
II	97 (41.99%)	0.54 (−0.19, 1.27)	0.15
III	104 (45.02%)	0.98 (0.26, 1.71)	**< 0.01**
NLR	2.98 ± 3.76	0.13 (0.07, 0.19)	**< 0.01**

*Note:* Data are presented as *n*(%) or mean ± standard deviation (SD) unless stated otherwise.

Abbreviations: BMI = body mass index, CHD = coronary artery disease, NLR = neutrophil-lymphocyte ratio, PP = pulse pressure.

**Table 4:** Multivariate linear regression of relationship between NLR and PLOS.

	**Model I**		**Model II**		**Model III**	
	**β** ** (95% CI)**	**p** **-value**	**β** ** (95% CI)**	**p** **-value**	**β** ** (95% CI)**	**p** **-value**
NLR	0.13 (0.07, 0.19)	**< 0.01**	0.11 (0.05, 0.17)	**< 0.01**	0.12 (0.06, 0.18)	**< 0.01**

*Note:* Model I: crude model; Model II: adjusted for gender, age, stature, weight, BMI, systolic pressure, diastolic pressure, pulse pressure, hypertension, diabetes, CHD, cerebral infarction, drank, and smoke; Model III: model II additionally adjusted for operation method, embolization, and Shamblin classification.

**Table 5:** Stratification analysis of associations between NLR and PLOS.

	**Model I**		**Model II**		**Model III**	
	**β** ** (95% CI)**	**p** **-value**	**β** ** (95% CI)**	**p** **-value**	**β** ** (95% CI)**	**p** **-value**
Shamblin I	0.06 (−0.03, 0.15)	0.21	0.03 (−0.07, 0.13)	0.59		
Shamblin II	0.20 (0.10, 0.31)	**< 0.01**	0.16 (0.08, 0.24)	**< 0.01**	0.20 (0.09, 0.29)	**< 0.01**
Shamblin III	0.15 (0.05, 0.25)	**< 0.01**	0.07 (−0.04, 0.19)	0.19	0.09 (−0.02, 0.19)	0.12

*Note:* Model I: crude model; Model II: adjusted for gender, age, stature, weight, BMI, systolic pressure, diastolic pressure, pulse pressure, hypertension, diabetes, CHD, cerebrovascular disease, drank, and smoke; Model III: model II additionally adjusted for operation method and embolization.

## Data Availability

The data that support the findings of this study are available from the corresponding authors upon reasonable request.
